# 33. Demyelination as a presenting feature of systemic lupus erythematosus: is it lupus or multiple sclerosis?

**DOI:** 10.1093/rap/rky033.024

**Published:** 2018-09-20

**Authors:** Holly Merris, Sofia Tosounidou

**Affiliations:** Rheumatology, Sandwell and West Birmingham NHS Trust, Birmingham, UNITED KINGDOM

**Introduction:** The differential diagnosis for the various manifestations of neuropsychiatric lupus are vast and sometimes complex to determine. This case of a young lady presenting with central nervous system demyelination, with a pattern typical of multiple sclerosis (MS), and concomitant features of acute systemic lupus erythematosus (SLE) demonstrates this diagnostic challenge. We present an overview of over ten years of this patient’s clinical history along with the current uncertainties for the patient and clinicians.

**Case description:** In late 2007 a 24 year old Afro-Caribbean lady born and brought up in the UK presented with a self-resolving episode of left leg numbness extending to the torso with a sensory level of T5. Symptoms had resolved by first contact with the neurology team, where presence of a subtle unilateral left optic neuritis was detected. The presentation of two clinically distinct episodes of neurology separated in time in this young lady strongly suggested the diagnosis of MS. An MRI performed in the following weeks showed distinct white matter lesions situated in a peri-ventricular distribution, features further characteristic of MS. The patient made good spontaneous neurological recovery initially, lending to the hypothesis that this was a relapsing-remitting MS. Screening serology performed by the neurology team unexpectedly revealed a high titre of ANA at 1: 1600 with anti-Ro positivity (dsDNA 86.2 mmol/L (<15), C3 0.70 mmol/L (0.75-1.75), C4 0.09 mmol/L (0.14 - 0.54), and negative antiphospholipid screening. She was referred to rheumatology where further evaluation revealed a history of widespread joint pains, swelling of fingers, photosensitive rash, Raynauds and sicca symptoms. She remained neurologically asymptomatic, with mild features of SLE until 2009 when she developed a spinal relapse of her disease. At this stage clinical examination demonstrated bilateral leg paraesthesia to waist level, incontinence, loss of vibration and proprioception sense in the lower limbs and Romberg’s sign positivity. This was treated with intravenous methylprednisolone with only partial success. This relapse marked the beginning of multiple recurrent episodes of demyelination with ongoing serological evidence of active SLE (hypocomplementaemia and significant titres of dsDNA) with a gradually decreasing clinical response to intravenous methylprednisolone infusions. By 2010, brain and spinal relapses were occurring regularly and failing to respond to treatment. CSF examination at this point showed the presence of oligoclonal bands - a feature not specific to MS. She continued to demonstrate active clinical and serological features of SLE. MRI lesions were periventricular, in keeping with the typical distribution of MS imaging. Intensive multidisciplinary discussion between neuroradiology, neurology and rheumatology and despite uncertainty, the decision was made to treat this as SLE associated demyelination. By 2011, clinical deterioration had continued and cyclophosphamide was used with some success followed closely by rituximab. After two courses of rituximab there was significant recovery neurologically, and serologically along with improvement in this young lady’s extra-cranial symptoms of SLE. She remained relatively asymptomatic neurologically for a period of time. Inflammatory arthritis symptoms were controlled with methotrexate and low doses of prednisolone. In late 2017, the patient developed left sided weakness, sensory loss and poor coordination. This was not associated with any worsening features of SLE: inflammatory arthritis remained quiescent, as did SLE serology. MRI examination at this point suggested further demyelination with typical imaging features of MS and high dose intravenous methylprednisolone was commenced once more. As of time of writing, discussion continues between neurology and rheumatology as to whether to attempt a further course of rituximab or to consider disease modifying therapy for MS with teriflunomide.

**Discussion:** At this stage in her management it is still not clear whether her disease can all be explained by SLE or whether she is affected concurrently with SLE and MS. It is possible that rituximab and cyclophosphamide would have controlled either auto-immune aetiologies. One of the clear lessons to learn from this scenario is that the when the aetiology is unclear, the safest clinical course can be to treat as SLE. The use of therapies such as interferon-beta for MS is known to worsen SLE, whereas the management strategies used for the control of neuropsychiatric SLE may also have a beneficial role in the management of patients where MS is an equally likely differential. SLE related demyelinating disease is rare, likely affecting at most 1% of patients with a diagnosis of SLE. However, SLE and MS are both known to have a propensity to affect a similar demographic, commonly women of childbearing age. Thus differentiating between the two disease entities in patients with a clinically isolated episode of demyelination and serology suggestive of SLE can prove difficult. There is no specific clinical, laboratory, or imaging test which will accurately differentiate SLE demyelination from MS, particularly when it may be the presenting feature of SLE. There are however a number of clinical, laboratory and radiological parameters that can lend support to one diagnosis over another. This includes serology, CSF examination, distribution of lesions on brain and spinal MRI, and the use of positron emission tomography (PET) and single-photon emission computed tomography (SPECT) scanning.

**Key Learning Points:** Demyelinating disease can rarely be a presenting clinical feature of SLE, or can complicate a pre-existing diagnosis of SLE. The diseases both tend to affect a similar demographic. Whilst differentiating between the two disease entities can be complex, there are a number of ways to help to determine the underlying disease aetiology. This can influence disease management. It is however still important to remember that some of the disease modifying strategies used in the treatment of MS can have adverse effect in those with SLE, and this may be the deciding factor that informs management.

**Disclosure: H. Merris:** None. **S. Tosounidou:** None.

